# GT-WGS: an efficient and economic tool for large-scale WGS analyses based on the AWS cloud service

**DOI:** 10.1186/s12864-017-4334-x

**Published:** 2018-01-19

**Authors:** Yiqi Wang, Gen Li, Mark Ma, Fazhong He, Zhuo Song, Wei Zhang, Chengkun Wu

**Affiliations:** 1 School of Computer Science, National University of Defense Technology, Changsha, 410000 China; 2 Genetalks Biotech. Co., Ltd, Beijing, 100000 China; 3 Department of Clinical Pharmacology, Xiangya Hospital, Central South University, Changsha, 410000 China

**Keywords:** Whole-genome sequencing, AWS, Parallel and distributed computing

## Abstract

**Background:**

Whole-genome sequencing (WGS) plays an increasingly important role in clinical practice and public health. Due to the big data size, WGS data analysis is usually compute-intensive and IO-intensive. Currently it usually takes 30 to 40 h to finish a 50× WGS analysis task, which is far from the ideal speed required by the industry. Furthermore, the high-end infrastructure required by WGS computing is costly in terms of time and money. In this paper, we aim to improve the time efficiency of WGS analysis and minimize the cost by elastic cloud computing.

**Results:**

We developed a distributed system, GT-WGS, for large-scale WGS analyses utilizing the Amazon Web Services (AWS). Our system won the first prize on the Wind and Cloud challenge held by Genomics and Cloud Technology Alliance conference (GCTA) committee. The system makes full use of the dynamic pricing mechanism of AWS. We evaluate the performance of GT-WGS with a 55× WGS dataset (400GB fastq) provided by the GCTA 2017 competition. In the best case, it only took 18.4 min to finish the analysis and the AWS cost of the whole process is only 16.5 US dollars. The accuracy of GT-WGS is 99.9% consistent with that of the Genome Analysis Toolkit (GATK) best practice. We also evaluated the performance of GT-WGS performance on a real-world dataset provided by the XiangYa hospital, which consists of 5× whole-genome dataset with 500 samples, and on average GT-WGS managed to finish one 5× WGS analysis task in 2.4 min at a cost of $3.6.

**Conclusions:**

WGS is already playing an important role in guiding therapeutic intervention. However, its application is limited by the time cost and computing cost. GT-WGS excelled as an efficient and affordable WGS analyses tool to address this problem. The demo video and supplementary materials of GT-WGS can be accessed at https://github.com/Genetalks/wgs_analysis_demo.

## Background

Whole-genome sequencing is prevalently used in research, and it has been increasingly popular in clinics in recent years [1–3]. WGS data plays an important role in guiding disease prevention, clinical diagnoses and therapeutic intervention [4, 5]. In some cases, WGS can help make clinical diagnoses that cannot be easily ascertained by conventional approaches [6, 7]. WGS by NGS is now transforming the diagnostic testing, treatment selection, and many other clinical practices, due to its ability to rapidly test almost all genes potentially related to diseases and help reveal the pathogenesis beneath symptoms, which is particularly meaningful in dealing with some rare or complex diseases. Moreover, with the proliferation of WGS, human genome databases are getting increasingly large. It provides researchers a great opportunity to conduct more comprehensive and profound genetic studies, with which we can better understand the relationship between genome and some complex diseases such as cancer and identify the effects of DNA variations.

In clinics, a rapid WGS analysis is urgent, because some diseases progress quickly and the sequencing rate potentially impact patients’ lives, and a timely diagnosis can help not only avoid futile therapies but find the most effective therapeutic interventions. Currently it usually takes 30 to 40 h to finish a 50× or deeper whole-genome sequencing task, which is far away from the demands of biotech industry. It is also of great necessity to make WGS less expensive, since only the price burden getting lower can more and more ordinary patients and researchers afford it and can WGS achieve further development and proliferation.

However, an efficient analysis of WGS data is not a trivial task, as it requires significant computing power and storage capacity. Table 1 [8–13] presents a comprehensive survey of previous benchmarks of WGS data analysis according to literature, with details on the time, cost, software pipeline and the hardware specification. As we can see, ‘BWA + GATK’ is one of the most popular pipelines. It’s difficult to compare the efficiency of those efforts on a completely fair base. Nevertheless, the average time and cost (42.9 h and $79.1) of all those works provide a general sense of the necessity to improve the efficiency of WGS analyses. Beside of dedicated hardware acceleration, such as GPU used in [13], cloud computing, which is a type of scalable and flexible computing infrastructure, is a prevailing solution for efficient sequencing data analyses [8, 9, 14, 15]. However, a few problems need to be addressed before a successful application. In a typical cloud environment, connectivity between nodes are usually not optimized for high performance computing, thus it is difficult to devise an adequately low-latency and high-bandwidth data transfer mechanism. Moreover, the potentially high price is also a huge obstacle hindering the further development and proliferation of WGS analyses on the cloud. Theoretically speaking, the running cost is proportional to the running time and the number of running nodes in cloud computing, which inevitably increases with the size of input datasets. Therefore, challenges for WGS applications by cloud computing are to fully leverage the infrastructure service, elastic scalability, and the billing strategy of cloud computing vendors. The key is to make a balance among storage, IO, computation and economic cost.

**Table 1:** Comparison with previous benchmarks of time and cost for WGS data analysis based on different pipeline and hardware

Tool	Aligner + Variant Caller	Depth	Time	Cost	Depth^a^	Time^a^	Cost^a^	Hardware
Genomekey + COSMOS [8]	BWA + GATK HaplotypeCaller	37×	4.9 h	$48.5	55×	7.3 h	$72.1	20× AWS c2.8xlarge
Churchill [8]	BWA + GATK UnifiedGenotyper	30×	1.7 h	–	55×	3.1 h	–	16× AWS r3.8×large
STORMseq [8]	BWA + GATK lite	38×	176 h	$32.8	55×	255 h	$47.5	–
Crossbow [9]	Bowtie + SOAPsnp	38×	4.5 h	$71.4	55×	6.5 h	$103.3	20× AWS c1.xlarge
Crossbow [9]	Bowtie + SOAPsnp	38×	2.5 h	$83.6	55×	3.6 h	$121	40× AWS c1.xlarge
PEMapper / PECaller [10]	PEMapper + PECaller	30×	29.3 h	–	55×	53.7 h	–	–
Globus [11]	Bowtie2 + GATK	30×	12 h	–	55×	22 h	–	1× AWS cr1.8xlarge
SevenBridges [12]	BWA + GATK	15×	8 h	$14.1	55×	29.3 h	$51.7	–
BGI-online (BALSA) [13]	BALSA	50×	5.5 h	–	55×	6 h	–	6-core CPU, 64GB RAM, GPU GTX680
Average						42.9 h	$79.1	

‘^a^’ means time and cost of different depth data are normalized to 55× with linear relationship. ‘-’ means not reported

## Results and discussion

GT-WGS, the distributed whole-genome computing system we built, is based on the cloud computing platform provided by Amazon Web Service. It has a good extensibility and ability, which can automatically scale out the cluster size according to the computing demand, thus minimizing the computation time of sequencing data. Meanwhile, GT-WGS can apply for resources based on the dynamic price offered by spot instances of AWS, consequently minimizing the computing expense. We used the 55× whole genome dataset (NA12878) provided by GCTA challenge committee and 500 5× whole-genome data provided by the XiangYa hospital to evaluate the computing efficiency and the economic efficiency of GT-WGS. According to the results, it took GT-WGS about 18 min to accomplish the 55× WGS data analysis at a computing cost of $16.5, and 2.39 min for each 5× whole-genome sequencing with $3.62 on average, and the output accuracy is up to standards required by the GCTA challenge.

### Results of 55× WGS data analyses

In this test, we utilized 300 machine instances altogether in the AWS eastern American computing center, including 250 m4.4×.large instances (for computation) and 50 r3.8×.large instances (for distributed file system). Such a configuration is empirically determined based on our experience and a number of tests. Firstly, the analysis efficiency is not proportional to the number of instances, as an increasing number of nodes would extra overhead in distributing computation. Hardware details of m4.4×.large instances and r3.8×.laerge instances are list in Table 2 (https://aws.amazon.com/ec2/instance-types). We also made use of the Amazon Simple Storage Service (Amazon S3) as the data storage system. The WGS data used (400GB NA12878) is provided by the GCTA committee. During the testing process, GT-WGS dynamically applied for spot instances, so as to minimize expenditure to the best extend.

**Table 2:** The configuration information of r3.8xlarge and m4.4×large

Instance Type	vCPU	Memory (GB)	Storage (GB)	Networking performance	Physical processor	Clock speed (GHz)
r3.8xlarge	32	244	2 × 320 SSD	10 Gigabit	Intel Xeon E5–2670 v2	2.5
m4.4xlarge	16	64	EBS Only	High	Intel Xeon E5–2676 v3	2.4

It took GT-WGS 18.4 min to finish the analysis, and the overall cost was $16.5: (250*$0.1287 + 50*$0.4386) *(18.4 mins/60mins). To note, costs of different machine instances vary. Details about the AWS cost and the overall time cost are illustrated in Table 3. The WGS analysis includes four major steps: mapping, BAM merging and sorting, variants calling and VCF merging, which took 4.7 min, 3.6 min, 8.9 min and 23.2 s respectively in our test, as described in Table 4.

**Table 3:** Time cost and AWS expenditure for 55× WGS

Overall time for the 55× WGS	Cost per m4.4×.large instance	Cost per r3.8×.large instance	Overall expenditure for the 55× WGS
18.4 min	$0.1287	$0.4386	$16.50

**Table 4:** Time cost for each step in 55× WGS

	Step	Time cost
1	Mapping	4.7 min
2	BAM Merging and Sorting	3.6 min
3	Variants calling	8.9 min
4	VCF Merging	23.2 s

For the 55× whole-genome sequencing data (NA12878) provided by GCTA committee, there are 4,073,208 single nucleotide polymorphism (SNP) mutation sites and 824,872 insert-deletion (Indel) mutation sites detected by GT-WGS in total. According to Table 1, the Churchill tool based on the Burrow-Wheeler Aligner (BWA) [16] and the GATK HaplotypeCaller [17] is the fastest cloud based solution [8]. It took 1.7 h (104 min) using 16× AWS r3.8xlarge instances (16*32 = 512 cores) on 30× data. To note, the size of the 55× WGS data on the same sample can be estimate to be 1.83 times of the 30× data. Thus the analyzing time would be approximately 191 min (104*1.83) on the 55× data. Comparison of the time cost between it with GT-WGS is listed in Table 5. In the Table 5, GT-WGS employed 250 m4.4×.large computation instances (250*16 = 4000 cores). When using the same amount of CPU cores, the speed up of GT-WGS versus the Churchill solution is: (191/18.4)/(4000/512) = 1.33.

**Table 5:** Comparison of overall time cost between GT-WGS and Churchill

Method	Overall time (min)	Number of CPU Cores
*GT-WGS*	18.4	250*16 = 4000
*Churchill*	191	16*32 = 512

We also compared that the results of GT-WGS and BWA + GATK to ensure the reliability of GT-WGS. The consistency of results was about 99.9%. Comparison details are listed in Table 6, where the proportion represents the ratio of the number of the specific mutation sites to that of all the mutation sites in corresponding method. For example, the seventh column of Table 6 shows us the proportion of number of common SNP mutation sites to total amount of SNP mutation sites is 99.8877% in GT-WGS and 99.8751% in BWA + GATK.

**Table 6:** Results comparison between GT-WGS and BWA + GATK

Mutation type	Unique mutation sites of GT-WGS		Unique mutation sites of BWA + GATK best practice		Common mutation sites		Mutation sites with consistent position but different genotype	
	Number	Proportion	Number	Proportion	Number	Proportion	Number	Proportion
SNP	3928	0.10%	4443	0.11%	4,067,370	(99.89%, 99.88%)	643	(0.016%, 0.016%)
INDEL	646	0.08%	675	0.08%	823,871	(99.90%, 99.89%)	197	(0.024%, 0.024%)

**Fig. 1:**
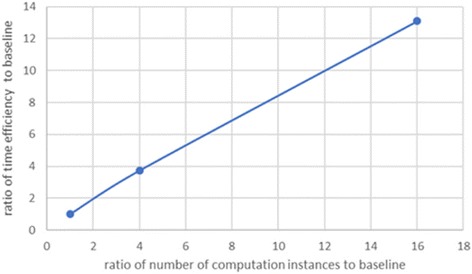
Speedup of GT-WGS

We further demonstrate the speedup of GT-WGS by utilizing different numbers of computation instances on the 55× whole-genome sequencing analysis. According to the results, the running time for the cases of 4, 16, 64 and 250 m4.4xlarge instances were 888.7, 238, 67.9, and 18.4 min respectively. It shows an almost linear speedup. Details of time overhead are demonstrated in Table 7, and Fig. 1 shows the speedup trend line of GT-WGS, where the performance of the case of 4 computation instances is regarded as the baseline.

**Table 7:** Results comparison among cased of different number of computation instances

	Number of computation instances (m4.4xlarge)	Time cost
1	4	888.7 min
2	16	238.0 min
3	64	67.9 min
4	250	18.4 min

### Results of 500 5× WGS data samples

We also did experiments on a SNP dataset from the XiangYa hospital, which is 12.6 TB in total and it consists of 500 5× whole-genome data samples. In this test, we used 250 r4.4xlarge instances, each of which cost $0.24 per hour, and 50 r3.8xlarge instances, each of which cost $0.61 per hour. (details shown in Table 8). The instance types used in different cases are determined by our system automatically, GT-WGS here chose r4.4xlarge instances because it was cheaper than m4.4xlarge while performing the evaluation and it has almost the same computing capability with that of m4.4xlarge. The specific configuration information of r4.4xlarge instances and r3.8xlarge instances can be seen in the Table 9. Load balancing and dynamic scheduling were exploited in this experiment to optimize resource utilization. For each 5× sample, it took 1.80, 0.40, 5.06 and 0.56 min respectively to finish mapping, BAM merging and sorting, variants calling and VCF calling. The average time cost for each 5× WGS analysis was 2.39 min and the average expenditure was $3.62. Table 10 describes time cost details in this case.

**Table 8:** AWS expenditure for 5× WGS

Cost per r4.4×.large instance	Cost per r3.8×.large instance	Overall expenditure for 500 5× WGS	Average expenditure for 5× WGS
$0.24	$0.61	$1810.0	$3.62

**Table 9:** The configuration information of r3.8xlarge instance and r4.4xlarge instance

Instance type	vCPU	Memory (GiB)	Storage (GB)	Networking performance	Physical processor	Clock speed (GHz)
r3.8xlarge	32	244	2 × 320 SSD	10 Gigabit	Intel Xeon E5–2670 v2	2.5
r4.4xlarge	16	122	EBS Only	Up to 10 Gigabit	Intel Xeon E5–2686 v4	2.3

**Table 10:** Time cost portfolio for 5× WGS time cost on average and in total

	Step	Time cost	Total time	Time per 5× WGS
1	Mapping	1.80 min	1199.74mins	2.39mins
2	BAM Merging and Sorting	0.40 min		
3	Variants calling	5.06 min		
4	VCF Merging	33.6 s		

GT-WGS is the champion solution of the WGS time optimization problem on the Wind and Cloud challenge held by the GCTA committee (see https://tianchi.aliyun.com/mini/challenge.htm for the news report). This success can be attributed to:

1. GT-WGS always tries to get the lowest price via spot instances;
2. GT-WGS makes full use of the computing resources with cleverly designed parallel processing techniques;
3. GT-WGS minimizes time waste through its load balancing and dynamic scheduling strategy.

GT-WGS can finish a typical analysis of one 5× WGS data sample within 3 min for less than $4. In our opinion, this could be a milestone in the biotech industry. GT-WGS is also effective for WGS data with higher depths. GT-WGS was able to process the 55× WGS dataset offered by the GCTA committee at a cost of $16.5 within 18.4 min (in the best case among all our tests). To note, a fluctuation of cost and computation time is inevitable due to the following two reasons: (1) we utilized the dynamic pricing of AWS to pursuit cheapest instances; (2) computing instances are virtual machines and several instances can be running on a same actual server, so if the AWS infrastructure is busy, then performance of instances could be affected. The worst case happened on Friday, which is usually the busiest working day of AWS (with the highest unit computing price), the expenditure of one 55× WGS analysis using GT-WGS is no more than $29 within 22 min.

The main reason why GT-WGS can reduce computing cost is that it makes good use of the dynamic pricing provided by AWS. However, it is not a trivial task to utilize these resources, since once the real-time bidding price is higher than the users’ payment, all the instances will be taken back. This rule calls for a carefully designed mechanism for fault tolerance. GT-WGS addresses the problem by storing each data block in 4 nodes (1 host node +3 backup nodes), which can avoid single-node failures of storage. Moreover, GT-WGS strictly restricts the data input size of each worker task so that it can be finished within a short time and any failure of a single worker won’t damage the whole computing process. In addition, GT-WGS also addresses the problem of a reasonable partition of input data to improve data transmission efficiency in a distributed environment and breaks down the two IO walls by novel strategies.

## Conclusions

In this paper, we developed a distributed WGS computing system based on Amazon Web Services (AWS) named GT-WGS. GT-WGS won the first prize on the Wind and Cloud challenge held by the Genomics and Cloud Technology Alliance conference (GCTA) committee. It took only 18.4 min for GT-WGS to finish the 55× whole WGS data analysis designated by GCTA committee in the best case, at a cost of $16.5. In addition, GT-WGS is also featured by its scalability on the cloud via load balancing and dynamic scheduling. We conducted a large-scale analysis of 500 5× WGS data samples by load balancing and dynamic scheduling in GT-WGS, and the average time cost for each sample was 2.39 min and the average price was $3.62.

GT-WGS can possibly open novel chances for biotech industry, where individuals can have their WGS data analyzed at a low price in a short time. The GT-WGS service will be publicly available in the form of RESTful APIs in the near future.

## Methods

The main purpose of our work is to perform WGS data analyses efficiently and economically. By reasonably breaking down WGS into smaller tasks and executing them in parallel, we can reduce the wall time of WGS analyses effectively. To avoid an increase in the computing expenditure brought about by extra overhead for parallel processing, we developed a smart strategy that can fully take advantage of the flexible pricing provided by Amazon Web Service (AWS) and its unique computing-resources usage pattern. The pricing information of AWS can be found at https://aws.amazon.com/ec2/pricing (accessed on April 5, 2017).

### Pay a lower price through spot instances

There are four types of Amazon Elastic Compute Cloud (Amazon EC2) instances: On-Demand, Reserved Instances, Spot Instances and Dedicated Hosts. Users can increase or decrease their compute capacity according to the real-time demand of their applications with On-Demand instances, and pay at the specified hourly rate. Dedicated Hosts provide users with physical EC2 servers dedicated for their uses, while Reserved Instances offer a capacity reservation by assigning reserved instances to a specific zone. In fact, numerous users who choose Reserved Instances need not to occupy the resources at all time, thus AWS provides spot instances, which allows users to bid on spare Amazon EC2 computing capacity, at a varying discount up to 90%. This dynamic pricing mechanism offers users a rather cheap price, but it has a very high demand for application stability, since there exists a risk for the instances being revoked at the end of any timing cycle. To make use of these cheap resources steadily, we take advantage of a high-tolerant system strategy, and make an elaborate design and limitation on the data volume and computing time of each unit task, which makes sure that there is no long-term task throughout the whole computing process, thus avoiding a huge loss when the system has to release resources.

### Strategies to improve computing efficiency

**Fig. 2:**
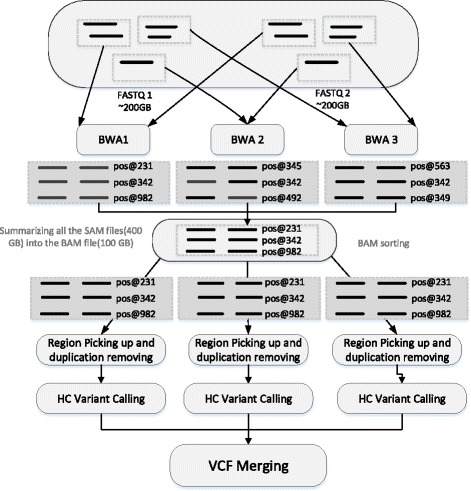
Two IO walls in the process of distributed WGS

In this paper, we aim to improve the computing efficiency of WGS analyses via proper parallel processing techniques. WGS data analyses usually include four major steps, but here we only discuss the first three steps since the time cost of the last step is much lower than others: mapping, BAM merging and sorting, and variants calling. WGS datasets are usually huge, especially in the mapping step and haplotype calling. We mainly focus on making a reasonable data partitioning and improving data transmission efficiency in a distributed computing environment. As seen in Fig. 2, we divide each FASTQ file into several parts, and assign them to corresponding BWA machines; then we gather all the SAM sequence alignment files into a sorted BAM file; next we assign the BAM file to different machines, pick up proper reads and perform HC variant calling and finally comes VCF merging. However, there exist two huge IO walls in such a computing process. The first IO wall exists in the data partition and transmission of the original FASTQ file, size of which is up to 400GB. Given the original file is distributed by one machine to many other machines, the assignment time is unacceptable even though the data transmission speed can be as fast as 1GB per second. The situation remains awful even if we alter the data distribution mode from one-to-many to many-to-many, since it costs a large amount of time to build up a distributed storage system required by many-to-many data assignment. The second IO wall appears when BWA mapping is done, all the SAM files produced by BWA computing nodes must be combined into one file, and then to be sorted, partitioned and distributed again to different nodes. To break down the two IO walls, we have designed and implemented StageDB, a hierarchical distributed database, also we built up an access interface based on it, which is consistent with POSIX file interface. These make up a data distribution system, which is capable of supporting random data block fetches simultaneously by hundreds of machines. This system partitions FASTQ files downloaded from S3 into many blocks, and stores them in one host node and three back-up nodes, which guarantees fast read speed even if several readers are reading the same data block simultaneously. For the second IO wall, we employ a multistage multi-node assigning and sorting method, and adjust the order of partition and assignment. Firstly, we partition the output of BWA mapping into several regions, and send them to the corresponding nodes. Next, sorting is done inside nodes, after that the sorting result is divided into hundreds of small-region files, which are then uploaded to AWS S3 system and transferred by S3 to the subsequent computing nodes. By hierarchical sorting and partitioning, plus adequate bandwidth and small-file parallel storage capacity provided by S3, we can break down the second IO wall perfectly.

### System architecture

**Fig. 3:**
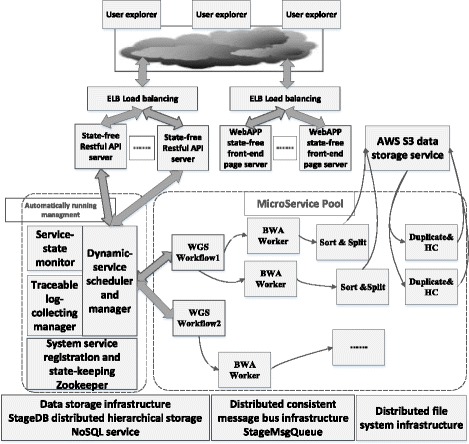
GT-WGS architecture

**Fig. 4:**
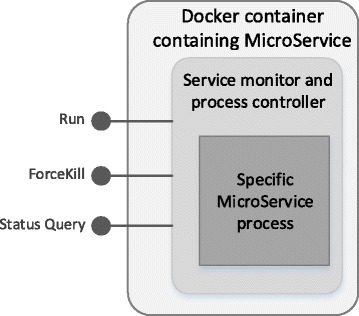
Structure of MicroService

As illustrated in Fig. 3, the system architecture is designed in a micro-service mode. Clients connect the backend of cloud computing system through restful interface to obtain computing states and data dynamically. In GT-WGS, we need to consider the following aspects: the ability to adjust computing capacity based on real-time users’ requirements; the ability to maximize computing efficiency with a low price; high tolerance of services drop-out and re-allocation and high robustness. As Fig. 4 shows, each micro-service is encapsulated by a basic service monitoring agent. The system manages and schedules micro-services by manipulating unified interfaces, regardless of the runtime details inside micro-services. In order to meet the demand of high dependability, each micro-service in GT-WGS must be able to tolerant arbitrary drop-outs of its dependent services, and regain stability via sophisticated state-transferring protocols. Apart from the distributed file system, all other software facilities are developed in-house, such as StageDB and StageMsgQueue. StageDB is a hierarchical distributed storage database, which offers services on the basis of hierarchy and whose services are far better than that of SQL database. StageMsgQueue is a persistent message-queueing service, which is capable of providing steady message persistence and queue notices.

**Fig. 5:**
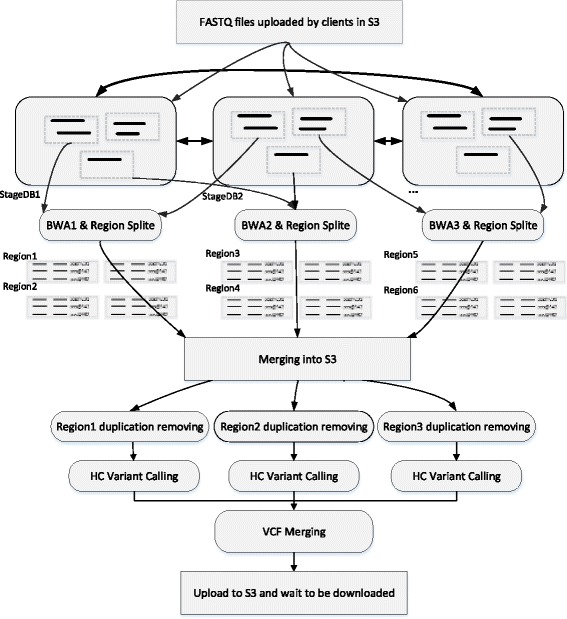
WGS analyzing process of GT-WGS

Figure 5 demonstrates the whole computing process of GT-WGS. We download FASTQ files from AWS S3, and store them into the data storage and distribution system based on StageDB. StageDB is deployed on 50 r3.8xlarge instances with high bandwidth, and the total output bandwidth is up to 50 GB per second. Next, BWA workers directly fetch data blocks from the distributed data-dispatching system and execute computing tasks. There are 250 BWA workers in total involved in our practice. The results from BWA workers are sorted by a region splitter, and then classified into hundreds of small region files. This kind of storage and merging mode fits the AWS S3 storage system well. Next, working nodes download computing data from corresponding regions, do sorting and remove unnecessary duplication. After that comes HC variant calling. Once all the HC computation is done, GT-WGS collects all results and uploads them to S3, where clients can download their final WGS result.

### Load balancing and dynamic scheduling

**Fig. 6:**
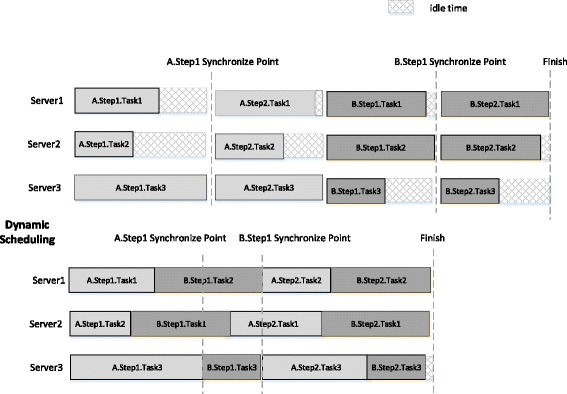
Dynamic task scheduling

There are lots of small tasks in each step of WGS analysis, and at the end of every step there exists a synchronization point among nodes, which means only when all the small tasks in the same step of different nodes finished, could the analysis process move into the next step. Time of different small tasks varies; thus, it is vital to introduce effective dynamic task scheduling in one node. A proof-of-concept illustration can be seen in the Fig. 6. In addition, we need to ensure the load balancing among different nodes, to reduce the waiting time among nodes in each synchronization.

## Abbreviations

Amazon EC2: Amazon Elastic Compute Cloud
Amazon S3: Amazon Simple Storage Service
AWS: Amazon web service
BAM: Binary alignment/map format
BWA: Burrow-Wheeler Aligner
DNA: Deoxyribonucleic acid
GATK: Genome Analysis Toolkit
GCTA: Genomics and cloud technology alliance conference
IO: Input/output
NGS: Next-generation sequencing
POSIX: Portable operating system interface of UNIX
SAM: Sequence alignment/map format
SNP: Single nucleotide polymorphism
SQL: Structured query language
VCF: Variant call format
WGS: Whole-genome sequencing
